# Social Mating System and Sex-Biased Dispersal in Mammals and Birds: A Phylogenetic Analysis

**DOI:** 10.1371/journal.pone.0057980

**Published:** 2013-03-06

**Authors:** Karen E. Mabry, Erin L. Shelley, Katie E. Davis, Daniel T. Blumstein, Dirk H. Van Vuren

**Affiliations:** 1 Department of Biology, New Mexico State University, Las Cruces, New Mexico, United States of America; 2 Department of Ecology and Evolutionary Biology, University of California Los Angeles, Los Angeles, California, United States of America; 3 Department of Biology and Biochemistry, University of Bath, Bath, United Kingdom; 4 Department of Wildlife, Fish, and Conservation Biology, University of California Davis, Davis, California, United States of America; University of Manitoba, Canada

## Abstract

The hypothesis that patterns of sex-biased dispersal are related to social mating system in mammals and birds has gained widespread acceptance over the past 30 years. However, two major complications have obscured the relationship between these two behaviors: 1) dispersal frequency and dispersal distance, which measure different aspects of the dispersal process, have often been confounded, and 2) the relationship between mating system and sex-biased dispersal in these vertebrate groups has not been examined using modern phylogenetic comparative methods. Here, we present a phylogenetic analysis of the relationship between mating system and sex-biased dispersal in mammals and birds. Results indicate that the evolution of female-biased dispersal in mammals may be more likely on monogamous branches of the phylogeny, and that females may disperse farther than males in socially monogamous mammalian species. However, we found no support for a relationship between social mating system and sex-biased dispersal in birds when the effects of phylogeny are taken into consideration. We caution that although there are larger-scale behavioral differences in mating system and sex-biased dispersal between mammals and birds, mating system and sex-biased dispersal are far from perfectly associated within these taxa.

## Introduction

Natal dispersal, the movement of individuals between their birthplace and site of first breeding, is crucial for a range of ecological and evolutionary processes [1], [2], [3]. Dispersal results in the redistribution of organisms and their genes, both within and between populations. Thus, dispersal influences processes as diverse as range expansions, population dynamics, and gene flow, to name but a few. Although the importance of dispersal is widely recognized, the process remains relatively enigmatic, largely due to the logistical difficulties that have historically hampered the study of dispersal in the field [1], [4].

One intriguing pattern that has long captured the attention of researchers is the fact that natal dispersal is often sex-biased within a species, with one sex dispersing further, or more frequently, than the other. In general, natal dispersal appears to be female-biased in birds (FBD), and male-biased in mammals (MBD), and empiricists and theoreticians have sought to explain this pattern for over 30 years [2], [5], [6], [7], [8], [9], [10]. Several non-mutually exclusive ultimate hypotheses have been put forward to explain the evolution of sex-biased natal dispersal, including inbreeding avoidance, competition for mates, and competition for resources [5], [6], [11], [12], [13]. Although most researchers recognize that there are likely multiple explanations for sex-biased dispersal in a given species [2], [12], debate about the relative importance of these factors continues today [10].

The most influential papers on sex-biased dispersal in birds and mammals are undoubtedly those of Greenwood [5] and Dobson [6], [14]. Greenwood proposed that in birds, which are typically socially monogamous and exhibit a ‘resource defense’ mating system, familiarity with local resources should be more important for males, who defend nests and territories, than for females, and predicted FBD in most birds (although the Family Anatidae, whose members tend to have ‘mate defense’ mating systems, has long been recognized as an exception to the pattern of FBD in birds). In contrast, many mammals are socially polygynous and display ‘mate defense’ systems, in which females rely on home ranges that contain the resources required to successfully rear offspring, and males mate with multiple females and often do not participate in the care of young. For most mammals, familiarity with local resources should be more important for females than males, and the expectation arises that males will disperse from their natal area [6]. In 1982, Dobson extended Greenwood’s ideas about divergent patterns of dispersal between birds and mammals to the relationship between mating system and sex-biased dispersal within mammals, and suggested that mating system may influence the direction of dispersal sex-bias, with equal or FBD dispersal being typical of monogamous mammalian species.

Greenwood and Dobson’s ideas about the relationship between mating system and sex-based dispersal in both birds and mammals have been widely accepted. However, there are at least two complicating factors that have not often been accounted for [10]. First, the lack of a standard definition of what constitutes ‘dispersal’ has led to confusion. Historically, dispersal has been most often quantified as either the proportion of individuals departing/disappearing from an area (sometimes defined as exceeding some minimum distance from the natal site), or as the straight-line distance between the natal and breeding locations of an individual. However, these two quantities measure different aspects of the dispersal process: the ‘departure’ definition assesses the initial decision of *whether to leave* the natal area at all, and the ‘distance’ definition assesses the subsequent decisions of *how far to travel* and *where to settle* once the initial departure decision has been made. Over the past decade, dispersal has become increasingly recognized as a multi-stage process consisting of three phases: departure from the natal area, searching for a new place to live, and settlement in the location where the animal will breed [1], [3], [15]. When we adopt this definition of dispersal, it becomes clear that the ‘departure’ and ‘distance’ criteria quantify behavior during different stages of the dispersal process. However, different authors use different definitions of dispersal, which has led to some confusion in the literature [10].

A second factor complicating the interpretation of the relationship between social mating system and sex-biased dispersal is the lack of a phylogenetic framework [2], [16], [17]. Greenwood and Dobson published their ideas in the early 1980s, well before phylogenetic comparative methods became widely available to behavior researchers. However, the behaviors observed in closely-related species cannot be considered independent data points because of the influence of shared ancestry [16], [18]. Thus, shared evolutionary history must be accounted for when examining correlations between behavioral traits across species. Surprisingly, the relationship between mating system and sex-biased dispersal in vertebrates has not yet been tested using modern phylogenetic methods [2], although Perrin & Mazalov [9] suggested over a decade ago that because similar dispersal patterns within taxa could be due to shared ancestry rather than mating system, analyses accounting for non-independence among species should be conducted.

Here, we attempt to resolve these complications by testing Dobson’s prediction that mating system should influence patterns of sex-biased dispersal, using a phylogenetic framework. We conduct separate analyses for four datasets: 1) Dobson’s original mammalian dataset, 2) the dataset compiled by Lawson Handley & Perrin in their 2007 review of sex-biased dispersal in mammals, 3) a newly-assembled dataset utilizing a quantitative measure of dispersal sex bias for mammals: relative dispersal distances for males and females within a species, and 4) a newly-assembled dataset quantifying relative dispersal distances for male and female birds. We examine each of these datasets in a phylogenetic framework, to determine whether social mating system is correlated with the direction of sex-biased dispersal, and whether the magnitude of the sex-bias is influenced by mating system. We focus on social, rather than genetic, mating systems for several reasons. First, the original hypothesis of a relationship between mating system and sex-biased dispersal was formulated before the ‘molecular revolution’ radically changed our notions about mating systems [19]. Second, despite rapid progress in determining genetic mating systems for both mammal and bird species, genetic mating system has not yet been quantified for enough species for which dispersal data are also available to make such an analysis possible. Third, to date, published studies indicate relatively little variation in broad categorizations of genetic mating system among species within either birds or mammals. The current literature on mammals illustrates these issues: genetic mating system data are available for fewer than half of the species included in our mammalian dispersal distance data set, and only 9.5% (2/21) of those species were genetically monogamous. Thus, genetic mating system data are yet sparse, and examples of rare mating systems (such as monogamy in mammals) are even more so.

An exhaustive review of either the support for various hypotheses to explain the evolution of social systems themselves or the evolution of sex-biased dispersal more broadly is beyond the scope of this study. Further, others have recently published reviews on both of these topics. We direct the interested reader to recent reviews of social evolution [20], [21] and sex-biased dispersal [2], [10] for more detailed information. Finally, we note that we do not attempt to test the relationship between mating system and sex-biased dispersal between birds and mammals, because a comparison based on just two taxa is impossible to test statistically.

## Materials and Methods

### Behavioral Data

We conducted phylogenetic analyses on four different datasets, three for mammals and one for birds. For mammals, we first reevaluated Dobson’s 1982 dataset [6], which categorized dispersal sex-bias using the proportion of dispersing individuals of each sex. We then conducted a phylogenetic analysis of the data presented in Lawson Handley and Perrin’s review [2], which includes studies of both the proportion of individuals of each sex dispersing and dispersal distances. Finally, we also compiled new datasets for both mammals and birds, in which we objectively quantified dispersal sex-bias using the ratio of dispersal distances between the sexes.

#### Mammals

We categorized mating systems as either socially monogamous (individuals form male-female pairs) or non-monogamous (‘non-monogamous’ encompassed all mating systems other than social monogamy, including polygyny/polygynandry). Mating systems other than monogamy and polygyny/polygynandry (e.g., polyandry) were too rare to warrant separate categorization. We categorized social mating system as monogamous or not monogamous, because these terms are more frequently reported in published studies than are mate defense vs. resource defense. We retained the author’s original categorization for both mating system and predominant dispersing sex for the reanalysis of existing datasets. When compiling novel datasets using dispersal distance, we again retained the mating system categorizations made by the authors, but calculated dispersal sex-bias using reported dispersal distances for females and males (described below). We also compiled a new dataset in which we categorized sex-biased dispersal using dispersal distance, rather than dispersal frequency. We searched the literature (using the key words “sex,” “dispers*,” “distance,” and “mammal*” in the Web of Knowledge database to identify species for which dispersal distance had been quantified) to obtain data on both social mating system and dispersal distance for mammal species. We identified 48 mammal species for which published data on both social mating system and a quantification of male and female dispersal distances were available (see Table S1). We only included field-based studies that quantified dispersal distance for each sex using direct methodologies such as resighting, live-trapping, radio-telemetry, or assigning offspring to parents using genetic methods. We did not include studies that reported a statistically significant effect of sex on dispersal distance, but no numerical summary of dispersal distance, or studies that reported a sex-bias in dispersal based on indirect genetic methods (such as spatial autocorrelation in genetic relatedness between males and females). We converted dispersal distances into a measure of sex-bias by taking the ratio of dispersal distance of females:males (quantified as the mean or median dispersal distance, depending upon data source). Because reported maximum dispersal distances are known to be subject to a detection bias [4], we never used maximum observed dispersal distance as the sole determinant of the direction of sex-biased dispersal. However, we did employ maximum dispersal distance to determine the direction of sex bias in one case in which the reported median dispersal distance was exactly the same for both sexes (*Dipodomys spectabilis*). The maximum dispersal distance confirmed that dispersal was female-biased in this species. Finally, for two species (*Martes pennanti* and *Odocoileus virginianus*), the direction of the sex-bias differed depending on whether mean or median dispersal distance was used to determine sex-bias. To be conservative, we conducted the analysis without these two species.

#### Birds

We compiled a dataset on sex-biased dispersal distance in birds using the same procedures described for the mammal dataset (above), sequentially replacing “mammal*” with “bird*” and “avian.” Our final dataset contained 56 bird species (see Table S2). Although previous authors have noted the predominance of female-biased dispersal in birds [5], [7], no one appears to have formally examined sex-biased dispersal in relation to mating system for birds (but see [8]), and there are no pre-existing datasets compiling information on both of these traits. We categorized social mating system and sex-biased dispersal distance for birds following the same procedures used for mammals (above).

### Phylogenies

#### Mammals

We used the species-level supertree published by Bininda-Emonds et al. [22] for the phylogenetic analysis. The mammal supertree contains 99% of extant mammalian species (4,510/4,554), with divergence times and branch lengths estimated using both fossil and molecular evidence. Although a recent study has challenged the divergence dates presented by Bininda-Emonds et al. [23], it does not present species-level data, and we retain the divergence dates presented by Bininda-Emonds. Using Mesquite 2.74 [24], we pruned the supertree to contain only the species for which we had behavioral data.

Modifications of the mammalian supertree were necessary to resolve polytomies and incorporate branch lengths for some species in the analysis of the Dobson and distance-based mammal data sets. In light of a reevaluation of the taxonomy of the ground squirrels that was published after the mammal supertree was compiled [25], we also modified the supertree to include updated topology and branch lengths for the ground squirrels (formerly all included within genus *Spermophilus*) and marmots [25], [26]. This modification was necessary given that ground squirrels make up a substantial proportion of Dobson’s dataset (14.5%: 8/55 species used in this study). We eliminated two species (*Psammomys obsesus* and *Redunca redunca*) because we were unable to determine their placement on the tree with branch lengths. The final tree for the reanalysis of Dobson’s data contained 55 species.

Similarly, we pruned the supertree to include only the species for which Lawson Handley and Perrin [2] reported mating system and dispersal data. We eliminated three species (*Microcebus berthae*, *Papio anubis*, and *P. cynocephalus*) from analysis because they were not included on the supertree. We also excluded from analysis *Homo sapiens* and *Microtus townsendii*, because the social mating systems reported for these species could not be cleanly categorized as either socially monogamous or not. The final tree for the phylogenetic analysis of this dataset contained 40 species.

We followed a similar procedure to obtain a fully resolved phylogeny with branch lengths for the species included in our mammalian dispersal distance dataset. We obtained the topology, branching patterns, and branch lengths for the ground squirrels from Harrison et al. [26] and Helgen et al. [25]. The supertree shows a polytomy for three species of *Microtus*, and we were unable to obtain a molecular phylogeny that included all three species. The *Microtus* polytomy was resolved through the removal of *M. townsendii*, which is not represented in a recent *Microtus* phylogeny [27]. There is disagreement between the supertree and a more recent phylogeny of the Carnivores [28] regarding the topology of the mustelids. We resolved the disagreement by removing *Mephitis mephitis*, the species whose placement was at issue. Removal of *M. mephitis* also eliminated a polytomy within the mustelids. The topology of the four species of *Peromyscus* included in this study is well-resolved, but not reflected in the supertree. Thus, we resolved the *Peromyscus* polytomy on the supertree according to published phylogenies [29], [30], [31], and scaled branch lengths using divergence time estimates provided by Jesse Weber (unpublished data). The final tree for this analysis contained 45 species.

#### Birds

We constructed a supertree from a subset of a large avian supertree dataset (Davis & Page, unpublished data) resulting in a dataset containing 56 taxa from 104 trees. These data were then checked for a sufficient level of taxon overlap (a minimum of two overlapping leaves between source trees). An MRP (Matrix Representation with Parsimony) matrix was then created and the analysis run in TNT [32] using the mult 30 = tbr drift command. The MRP analysis resulted in 234 MPTs (most parsimonious trees) of length 383. We computed the strict consensus tree for use in these analyses. All data processing was carried out using the Supertree Toolkit software package [33]. The final bird tree did not include branch length estimates; we simulated branch lengths using the option to export an ultrametric tree provided by Mesquite.

### Phylogenetic Analyses

**Figure 1:**
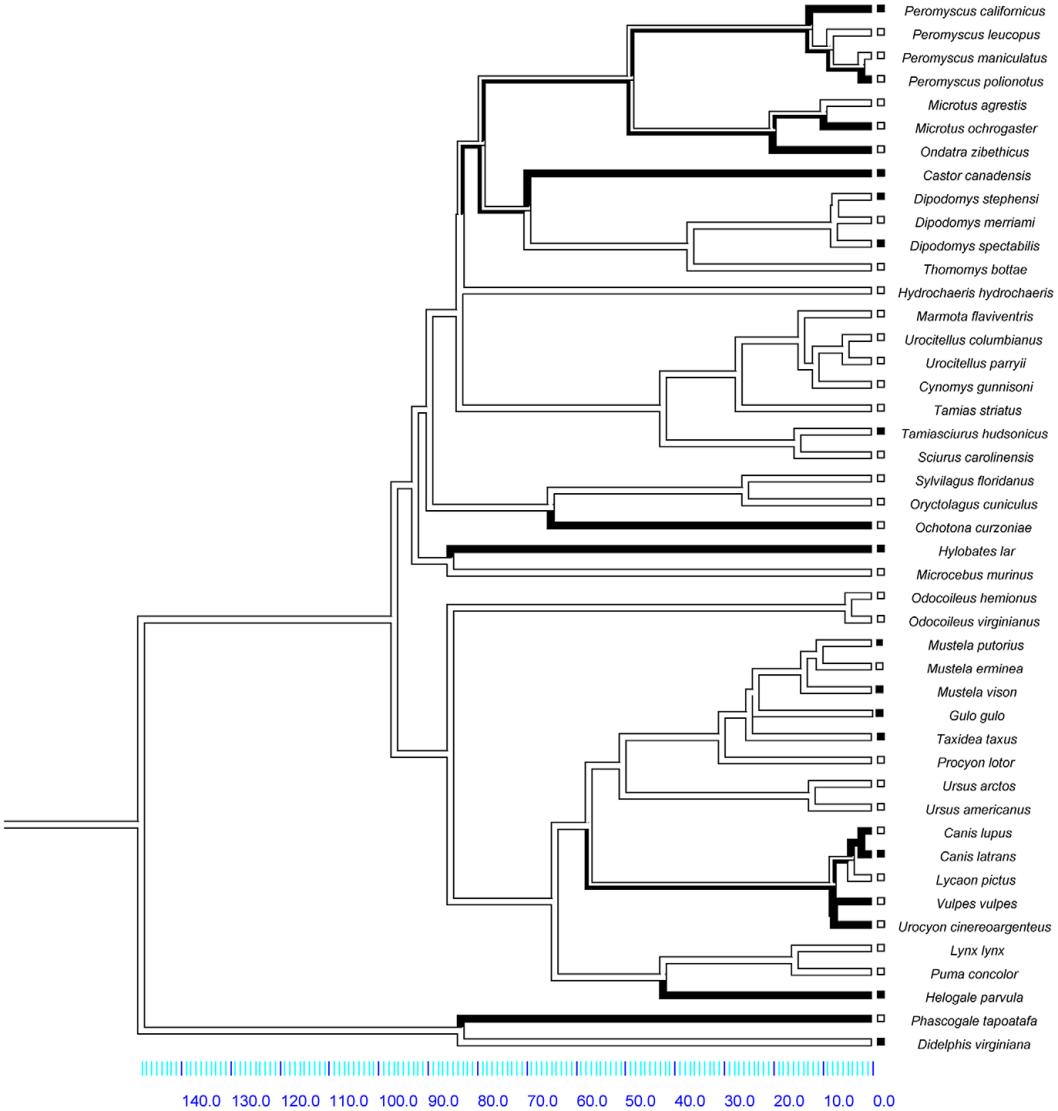
Phylogeny used for the analysis of the mammalian dispersal distance dataset. Time is shown in millions of years. Social mating system is traced over the branches; social non-monogamy (the ancestral state) is represented by white branches and social monogamy is represented by black branches. The boxes represent character states for sex-biased dispersal, with white boxes for species with MBD and black boxes for species with FBD.

**Figure 2:**
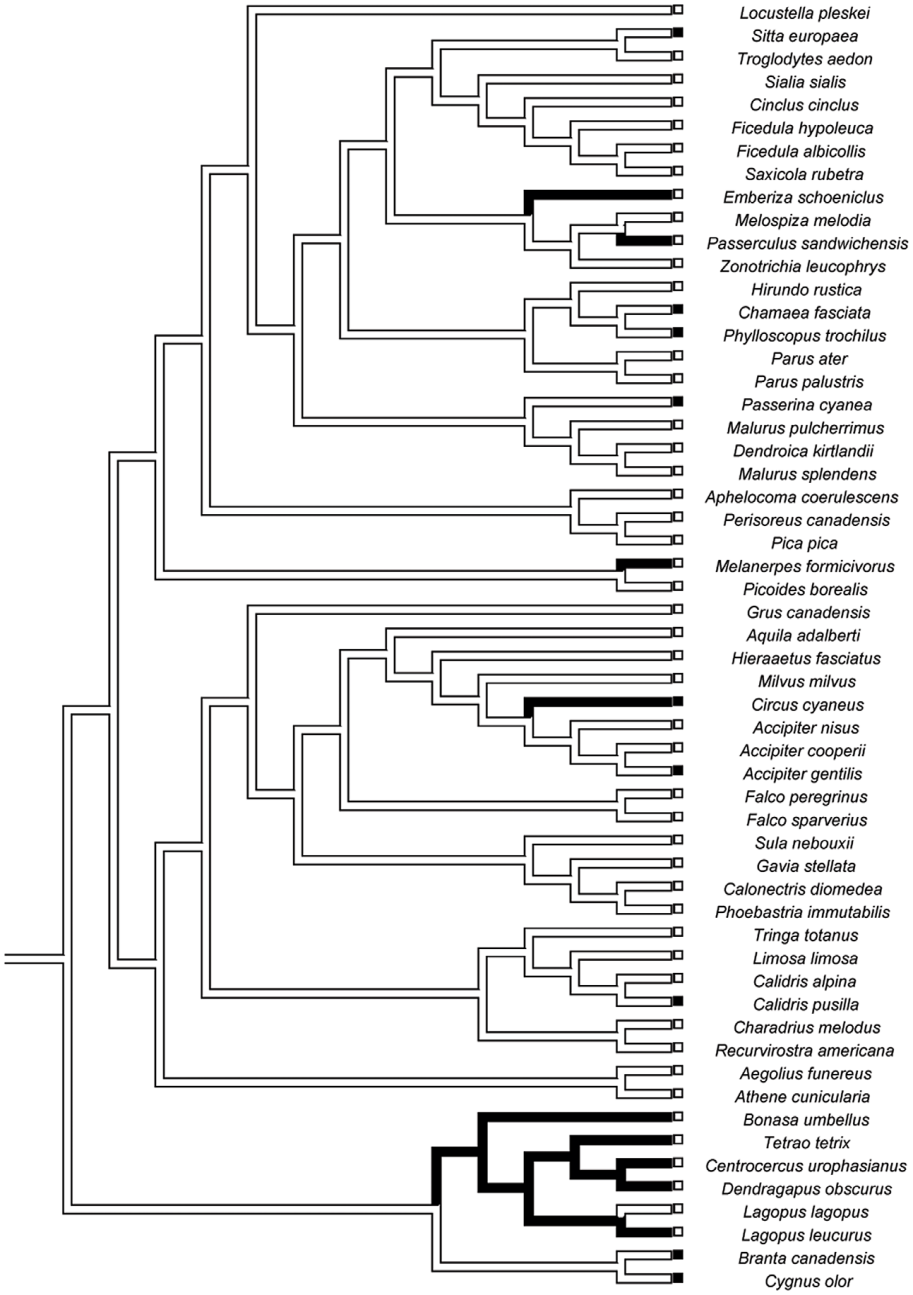
Tree used for the analysis of the avian dispersal distance dataset. Branch lengths are approximated. Social mating system is traced over the branches; social monogamy (the ancestral state) is represented by white branches, social non-monogamy is represented by black branches. The boxes represent character states for sex-biased dispersal, with white boxes for species with FBD and black boxes for species with MBD.

We determined the ancestral state for mating system and sex-biased dispersal separately for mammals and birds by parsimony in Mesquite 2.74 [24]. As expected, the ancestral condition for mammals was non-monogamy and MBD, and the ancestral condition for birds was monogamy and FBD (Figures 1–2).

#### Does mating system affect the likelihood of the evolution of a novel pattern of sex-biased dispersal?

We conducted tests for correlated evolution of two categorical traits: mating system and sex-biased dispersal, using the Discrete function in the program BayesTraits [34]. BayesTraits uses maximum likelihood methods to test specific models of correlated evolution and to estimate transition rates between four evolutionary states, using two traits, each of which has two possible values. For both mammals and birds, we coded ancestral character states as “0,” and derived character states as “1.” For rare instances in which the ratio of female:male dispersal distance was equal to 1 (i.e., reported dispersal distances for females and males were identical), we considered those species to possess the derived character state (FBD for mammals and MBD for birds), following Dobson’s prediction that in mammals, monogamy should be associated with female-biased or equal dispersal between the sexes. Because mammals and birds have different ancestral states for both social mating system and sex-biased dispersal, the four evolutionary states were coded as follows: mammals - 1 (non-monogamy/male-biased dispersal), 2 (non-monogamy/female-biased dispersal), 3 (monogamy/male-biased dispersal), and 4 (monogamy/female-biased dispersal); birds - 1 (monogamy/female-biased dispersal), 2 monogamy/male-biased dispersal), 3 (non-monogamy/female-biased dispersal), and 4 (non-monogamy/male-biased dispersal). Subscripts indicate the evolutionary states between which transition rates are estimated; for example, q_12_ indicates the transition rate from state 1 to state 2.

The central prediction made by the mating system/sex-biased dispersal hypothesis is that female-biased dispersal (or dispersal by both sexes) should be more likely to arise in socially monogamous than in non-monogamous mammal species [6]. Conversely, for birds, which have an ancestral state of social monogamy and FBD, the expectation is that male-biased dispersal should be more likely to arise in socially non-monogamous species. Following Pagel [35], we restricted our analysis to a single comparison between two models for each data set: a model of dependent evolution (all eight transition rates allowed to vary) and a model that restricted q_12_ = q_34_. We compared the dependent and restricted models statistically using a likelihood-ratio test, and then examined parameter estimates for transition rates.

The analytical method placed certain constraints on our ability to analyse some of the more subtle aspects of these datasets. For example, because we were limited to two states for each trait, we were unable to directly examine more complex situations such as that found in the Anatidae, a family of birds known to exhibit MBD [5], [7], [8]. It has been suggested that the resource-defense mating systems common in the Anatidae may explain the widespread occurrence of MBD in this group [5], [7]. Because we were unable to deal with this complexity any other way, we conducted the analysis both with and without two members of the Anatidae (*Branta canadensis* and *Cygnus olor*) included in our data set.

#### Does mating system affect the magnitude of the sex-bias in dispersal?

To examine whether social mating system influenced the magnitude of sex-biased dispersal, we used the PDAP module of Mesquite [36] to conduct phylogenetic generalized least squares (PGLS) analysis for both mammals and birds. For each test, we used the ratio of female:male dispersal distance as the response variable, and social mating system as the predictor. Branch lengths were log-transformed prior to analysis. This analysis was only possible for the two datasets that we compiled using dispersal distance, because the Dobson (1982) and Lawson Handley and Perrin (2007) datasets did not include dispersal distance, precluding the calculation of the ratio of F:M dispersal distance.

## Results

### Does Mating System Affect the Likelihood of the Evolution of a Novel Pattern of Sex-biased Dispersal?

#### Mammals

**Figure 3:**
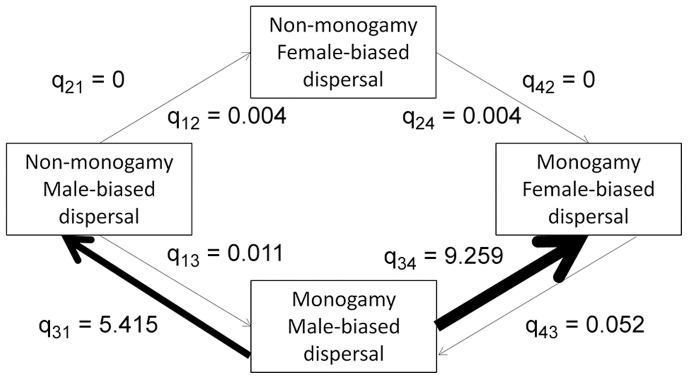
Path diagram showing evolutionary transition rates among four character states (Dobson’s dataset). Transitions from the ancestral state of non-monogamy and MBD to the derived state of monogamy and FBD for mammals using Dobson’s dispersal frequency based dataset are shown. Arrow thickness is proportional to the magnitude of the transition rate.

Proportions of monogamous and non-monogamous species exhibiting different patterns of sex-biased dispersal were presented by Dobson [6], who reported MBD in 78% of non-monogamous species and FBD in 92% of monogamous species. A likelihood ratio test (LRT) comparing the dependent and restricted models of evolution for this dataset was not statistically significant (χ*^2^_1_* = 0.008, *P* = 0.93). However, a comparison of transition rates estimated from the dependent model of evolution indicated that the evolution of FBD is >2000 times more rapid in socially monogamous than in non-monogamous species (q_34_ = 9.259> q_12_ = 0.004; Figure 3). Further, the character state of social monogamy and MBD appears to be unstable, with rapid transitions to either the ancestral state (non-monogamy and MBD) or the derived state for both traits (monogamy and FBD; Figure 3).

**Figure 4:**
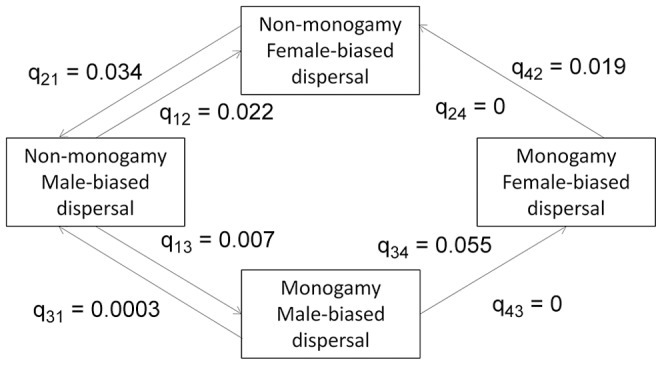
Path diagram showing evolutionary transition rates among four character states (Lawson Handley and Perrin’s dataset). Transitions from the ancestral state of non-monogamy and MBD to the derived state of monogamy and FBD for mammals using Lawson Handley and Perrin’s dataset are shown. Arrow thickness is proportional to the magnitude of the transition rate.

The Lawson Handley and Perrin dataset provides an interesting contrast to that of Dobson, because it focuses heavily on members of the Order Primates, whereas Dobson’s dataset contains many members of the Order Rodentia, and in particular, members of the former genus *Spermophilus*. Lawson Handley and Perrin report similar numbers of species with MBD (N = 25) and FBD (N = 21); however, it should be noted that they do not claim to have provided an exhaustive list of species, and have pooled multiple species within *Ateles* and the former genus *Spermophilus* into a single entry for each genus. These authors report MBD in 56% (22/39) of the non-monogamous species in their review, and FBD in 60% (3/5) of socially monogamous species (total N = 44). The results of the phylogenetic analysis of these data were not statistically significant (χ*^2^_1_* = 0.012, *P* = 0.91). The rate of evolution of FBD was ∼2.5 times greater on monogamous than non-monogamous branches (q_34_ = 0.055> q_12_ = 0.022; Figure 4).

Within the distance-based dataset, thirteen species were socially monogamous, and of those, five (38%) exhibited FBD, as predicted. However, 28% of non-monogamous species (9/32) also exhibited FBD (total N = 45). Seventy-two percent (23/32) of non-monogamous species exhibited MBD. Overall, the frequency of FBD appears to be slightly higher in socially monogamous mammals, but ‘mismatches’ between mating system and direction of the sex-bias in dispersal distance are not uncommon (Figure 1).

**Figure 5:**
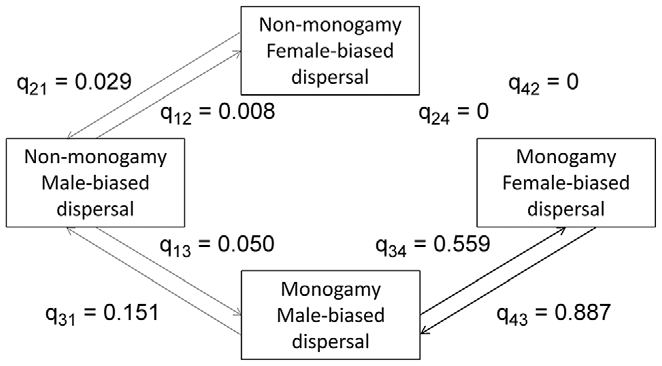
Path diagram showing evolutionary transition rates among four character states (mammalian dispersal distance dataset). Transitions from the ancestral state of non-monogamy and MBD to the derived state of monogamy and FBD for mammals using a dataset that quantifies sex-biased dispersal based on dispersal distances are shown. Arrow thickness is proportional to the magnitude of the transition rate.

Results of the phylogenetic analysis for mammalian dispersal distance were similar for all analyses, so we report only the results for the most conservative dataset, which eliminated all species about which there was uncertainty regarding the determination of the direction of sex bias (*Martes pennanti* and *Odocoileus virginianus*). The statistical comparison of the dependent and restricted models was not significant (LRT: χ*^2^_1_* = 0.112, *P* = 0.74). Although transition rates are generally low, parameter estimates from the dependent model show that FBD appears to be ∼70 times more likely to arise in socially monogamous species than in non-monogamous species (Figure 5; q_34 = _0.559> q_12_ = 0.008).

#### Birds

**Figure 6:**
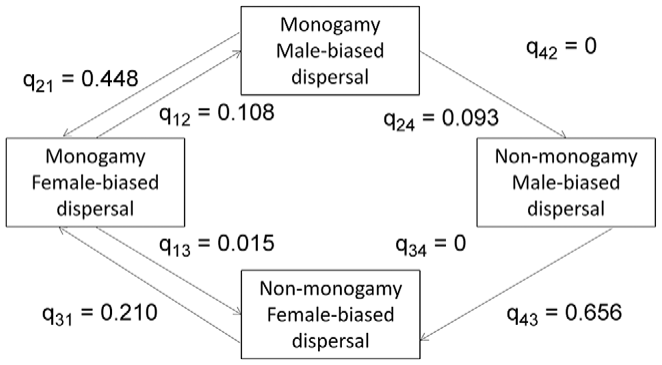
Path diagram showing evolutionary transition rates among four character states (avian dispersal distance dataset). Transitions from the ancestral state of monogamy and FBD to the derived state of non-monogamy and MBD for birds are shown. Arrow thickness is proportional to the magnitude of the transition rate.

Female-biased dispersal was observed in 39/47 (83%) of socially-monogamous birds. However, 89% (8/9) of non-monogamous species also exhibited FBD. Only 1/10 (10%) of non-monogamous species showed the expected pattern of MBD (Figure 6). Seventy percent (39/56) of bird species in this analysis exhibited the ancestral state: social monogamy and FBD.

Results were similar whether or not the Anatidae were included in the phylogenetic analysis. Thus, we report results for the full dataset. The Discrete comparison of the dependent and restricted models of evolution for birds was not statistically significant (Likelihood ratio test: χ*^2^_1_* = 0.024, *P* = 0.88). As for mammals, the mating system/sex-biased dispersal hypothesis predicts q_34_> q_12_ for birds. However, when we investigated parameter estimates from the dependent model of evolution, we found the opposite pattern: q_34_ = 0< q_12_ = 0.108 (Figure 6).

### Does Mating System Affect the Magnitude of the Sex-bias in Dispersal?

**Figure 7:**
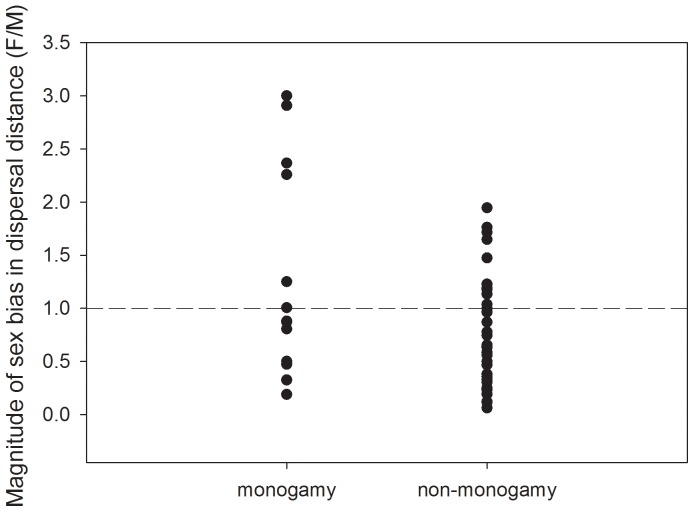
Magnitude of the sex-bias in dispersal distance in mammals, by mating system (socially monogamous or not).

We found evidence that females dispersed farther than males in socially monogamous mammalian species (PGLS: *F* = 5.55, *P* = 0.02, *r^2^* = 0.11; Figure 7). This pattern appeared to be strongly influenced by four socially monogamous species in which female dispersal distances were 2–3 times larger than male dispersal distances: *Helogale parvula* (F:M dispersal distance ratio = 3.00), *Castor canadensis* (ratio = 2.91), *Peromyscus californicus* (ratio = 2.37), and *Hylobates lar* (ratio = 2.26). However, we did not find evidence of a similar pattern in birds (PGLS: *F* = 0.24, *P* = 0.63, *r^2^* = 0.01).

## Discussion

Our results provide some support for a relationship between social mating system and sex-biased dispersal in mammals, but no support for such a relationship in birds. In both groups, the evolution of a derived state of sex-biased dispersal (FBD for mammals, MBD for birds) is a relatively rare event (Figures 1–2), and the vast majority of species exhibit the ancestral character state for both traits. There is some suggestion that within mammals, FBD is more likely to arise if a transition to social monogamy has already occurred (Figures 3,4,5), a result found across three datasets, including datasets that categorize the direction of sex-biased dispersal using both dispersal frequency and dispersal distance. However, only one bird species with a socially non-monogamous mating system exhibits MBD (*Circus cyaneus*), and socially monogamous bird species with MBD are scattered across the tree (Figure 6).

The magnitude of the sex-bias in dispersal distance appeared to be influenced by social mating system in mammals, but not in birds. However, the mammalian pattern appeared to be influenced by four species with female dispersal distances that were more than twice those of males (Figure 7). Of the ten remaining socially monogamous mammals in this analysis, nine had MBD, and one had approximately equal dispersal distances between the sexes, with a dispersal ratio of 1.01. Meanwhile, non-monogamous mammals showed variation in F:M dispersal distance ratio ranging from 0.06 to 1.95. Taken together, these results lead us to conclude that although there are clear differences in both social mating system and patterns of sex-biased dispersal between mammals and birds, there is currently insufficient evidence to determine definitively whether social mating system is causally responsible for variation in sex-biased dispersal within taxa. While we consider these results to be preliminary, they are generally in agreement with other recent studies of the influence of mating system and sociality on dispersal in mammals. For example, a recent review of dispersal in arvicoline rodents found no evidence of an influence of mating system on sex-biased dispersal [37].

Why did we find relatively limited support for a relationship between social mating system and sex-biased dispersal? One possibility is that each phylogenetic analysis included <60 species, which represents only a fraction of extant mammalian and avian species. Although larger sample sizes would be desirable, reliable estimates of dispersal distances for both sexes quantified through direct methods in natural populations in the field (e.g. telemetry, trapping, resighting) are difficult to obtain, a problem that has long been recognized [4]. Further, as molecular techniques for studying dispersal become more frequently used (reviewed by [2]), direct estimations of dispersal distances are becoming less common. For example, in our literature searches, we found only 11 new accounts of sex-biased dispersal in mammals published since 2000; a rate of about 1 species/year for the past decade. However, we did find consistent results across three different datasets compiled for mammals, which suggests that our findings are not spurious.

Given the relatively large number of ‘mismatches’ between social mating system and the predicted direction of the dispersal sex-bias in our data, it seems unlikely that the lack of strong correlations between these traits is due solely to sample size. For example, ≥20% of both mammal and bird species that possessed the ancestral mating system exhibited the derived character state for sex-biased dispersal. Further, 60% of mammals and 90% of birds possessing a derived mating system exhibited the ancestral state for sex-biased dispersal. We caution against extending the idea that ‘polygynous males’ and ‘monogamous females’ disperse to species in which dispersal behavior has not been quantified. The relationship between social mating system and sex-biased dispersal is more complex than has often been appreciated [2], [14], [37]. To date, most studies of the relationship between mating system and sex-biased dispersal have been conducted with mammals and birds. As mating system and sex-biased dispersal are quantified in fishes, amphibians, and non-avian reptiles [14], comparisons among additional vertebrate groups will be facilitated.

In the analyses presented here, we have only considered social mating system. When genetic mating systems are described for additional species, it will be interesting to see whether/how genetic mating system is related to sex-biased dispersal. For example, our mammalian data sets contain several socially monogamous mammal species that are known to be genetically non-monogamous, including some ‘model systems’ for the study of mammalian monogamy (e.g. prairie voles, *Microtus ochrogaster* [38]; beavers, *Castor canadensis* [39]). It remains to be seen whether incorporating genetic mating system data will clarify or further complicate our understanding of the evolutionary relationship between mating system and sex-biased dispersal in vertebrates.

## Supporting Information

Table S1 Mating system and dispersal data for mammals.
(PDF)

Table S2 Mating system and dispersal data for birds.
(PDF)
