# Supplementary material for: Social Mating System and Sex-Biased Dispersal in Mammals and Birds: A Phylogenetic Analysis
Source: PLoS One. 2013 Mar 6;8(3):e57980. doi: 10.1371/journal.pone.0057980 (PMC3590276; doi:10.1371/journal.pone.0057980)
Supplement: Table S1 — Mating system and dispersal data for mammals. (PDF) [file pone.0057980.s001.pdf]

Table S1: mating system and dispersal data for mammals.

taxonomy	Latin name	common name	social mating system	male dispersal distance (m)	female dispersal distance (m)	dispersal sex-bias	sources
Artiodactyla							
Cervidae	*Odocoileus hemionus*	mule deer	non-monogamy	15,200	12,200	male	Anderson & Wallmo 1984; Bunnell & Harestad 1983
	Odocoileus virginianus^	white-tailed deer	non-monogamy	18,500; 12,900*	19,500; 8,200*	-	Dusek et al. 1989
Carnivora							
Canidae	*Canis latrans*	coyote	monogamy	42,016*; 176,000#	42,250*; 232,200#	female	Andrews & Boggess 1978; Bekoff, 1977, Bowen 1982; Harrison 1992; Roy & Dorrance 1985
	Canis lupus	wolf	monogamy	154,000	123,000	male	Ballard et al. 1997; Gese & Mech 1991
	Lycaon pictus	African wild dog	non-monogamy	40,700*	19,000*	male	McNutt 1996; Nowak 1991
	Urocyon cinereoargenteus	grey fox	monogamy	16,000*	5,200*	male	Fritzelle & Haroldson 1982; Tullar & Berchielli 1982
	Vulpes vulpes	red fox	monogamy	29,002*	13,708*	male	Jensen 1973; Storm et al. 1976; Tullar & Berchielli 1980
Felidae	*Lynx lynx*	northern lynx	non-monogamy	60,250	7,000	male	Nowak 1991; Schmidt 1998
	Puma concolor	cougar	non-monogamy	37,300	11,300	male	Currier 1983; Maehr et al. 2002
Herpestidae	*Helogale parvula*	dwarf mongoose	monogamy	250*	750*	female	Nowak 1991; Rood 1987

Mustelidae	*Gulo gulo*	wolverine	non-monogamy	51,000	60,000	female	Vangen et al. 2001; Wilson 1982
	Martes pennant^	fisher	non-monogamy	10,800; 10,700*	11,200; 10,000*	-	Arthur et al. 1993; Nowak 1991
	Mephitis mephitis^	striped skunk	non-monogamy	3,000	2,900	male	Nowak 1991; Rosatte & Gunson 1984
	Mustela erminea	ermine	non-monogamy	1,986*; 5,600#	754*; 1,000#	male	Erlinge 1977; King 1983
	Mustela putorius	ferret	non-monogamy	6,700	11,800	female	Byrom 2002; Nowak 1991
	Mustela vison	mink	non-monogamy	18,024	26,553	female	Mitchell 1961; Nowak 1991
	Taxidea taxus	North American badger	non-monogamy	11,000*; 110,000#	13,500*; 52,000#	female (based on medians)	Lindzey 1978; Long 1973; Messick & Hornocker 1981
Procyonidae	*Procyon lotor*	raccoon	non-monogamy	9,700; 14,400*	600; 10,900*	male	Gehrt & Fritzell 1998; Stuewer 1943
Ursidae	*Ursus americanus*	American black bear	non-monogamy	61,000; 49,000*	7,333; 8,000*	male	Larivière 2001; Rogers 1987
	Ursus arctos	grizzly bear	non-monogamy	29,900; 5,500*; 134,000#	9,800; 9,500*; 82,000#	male	Glenn & Miller 1980; McLellan & Hovey 2001; Pasitschniak-Arts 1993; Pearson 1972
Dasyuromorphia							
Dasyuridae	*Phascogale tapoatafa*	brush-tailed phascogale	monogamy	5,800; 4,500*	1,100; 650*	male	Nowak 1991; Soderquist & Lill 1995
Didelphimorphia							
Didelphidae	*Didelphis virginiana*	Opossum	non-monogamy	1,661*	1,882*	female	Gillette 1980; Nowak 1991

Lagomorpha							
Leporidae	*Oryctolagus cuniculus*	European rabbit	non-monogamy	220	80	male	Nowak 1991; Richardson et al. 2002
	Sylvilagus floridanus	eastern cottontail	non-monogamy	54	42	male	Chapman et al. 1980; Chapman & Tretheway 1972
Ochotonidae	*Ochotona curzoniae*	plateau pika	monogamy	46	15	male	Dobson et al. 1998
Primates							
Cheirogaleidae	*Microcebus murinus*	grey mouse lemur	non-monogamy	251*	63*	male	Radespiel et al. 2003
Hominidae	*Hylobates lar*	gibbon	monogamy	620; 500*	1400; 1400*	female	Brockelman 1998; Leighton 1987
Rodentia							
Castoridae	*Castor canadensis*	beaver	monogamy	3,490; 1,990*	10,150; 6,060*	female	Jenkins & Busher 1979; Sun et al. 2000
Geomyidae	*Thomomys bottae*	Botta's pocket gopher	non-monogamy	81; 45*	53; 20*	male	Daly & Patton 1990; Nowak 1991
Heteromyidae	*Dipodomys merriami*	Merriam's kangaroo rat	non-monogamy	62*; 250#	50*; 158#	male	Jones 1989; Randall 1993
	Dipodomys spectabilis	banner-tailed kangaroo rat	non-monogamy	75*; 900#	75*; 2,000#	female or equal between sexes	Jones 1987; Jones et al. 1988; Randall 1993
	Dipodomys stephensi	Stephens' kangaroo rat	non-monogamy	30	39	female	Price et al. 1994; Randall 1993

Hydrochoeridae	*Hydrochaeris hydrochaeris*	capybara	non-monogamy	4,025[*]	3,500[*]	male	Herrera 1992; Herrera & McDonald 1993
Muridae	*Microtus agrestis*	field vole	non-monogamy	59; 159[#]	29; 194[#]	male	Nowak 1991; Sandell et al. 1990
	Microtus ochrogaster	prairie vole	monogamy	33; 23[*]; 136[#]	29; 18[*]; 127[#]	male	McGuire et al. 1993
	Microtus townsendii[^]	Townsend's vole	non-monogamy	19	12	male	Lambin 1994; Nowak 1991
	Ondatra zibethicus	muskrat	monogamy	65	41	male	Caley 1987; Errington & Errington 1937; Errington 1944; Willner et al. 1980
	Peromyscus californicus	California mouse	monogamy	41[*]; 450[#]	97[*]; 791[#]	female	Ribble 1992
	Peromyscus leucopus	white-footed mouse	non-monogamy	102[*]	57[*]	male	Jacquot & Vessey 1995; Keane 1990; Lackey et al. 1985
	Peromyscus maniculatus	deer mouse	non-monogamy	183[*]; 883[#]	107[*]; 1005[#]	male (based on medians)	Dice & Howard 1951
	Peromyscus polionotus	old-field mouse	monogamy	175	141	male	Swilling & Wooten 2002
Sciuridae	*Cynomys gunnisoni*	Gunnison's prairie dog	non-monogamy	198	126	male	Hoogland 1999
	Marmota flaviventris	yellow-bellied marmot	non-monogamy	1,825[*]	350[*]	male	Van Vuren 1990
	Sciurus carolinensis	eastern grey squirrel	non-monogamy	235[*]	175[*]	male	Cordes & Barkalow 1972
	Tamias striatus	eastern chipmunk	non-monogamy	345; 174[*]	85; 25[*]	male	Elliott 1978; Loew 1999
	Tamiasciurus hudsonicus	North American red squirrel	non-monogamy	253	301	female	Larsen & Boutin 1994; Wauters & Dhondt 1993
	Urocitellus columbianus	Columbian ground squirrel	non-monogamy	351[*]	338[*]	male	Hackett 1987 ; Murie & Harris 1984
	Urocitellus parryii	Arctic ground squirrel	non-monogamy	515; 241[*]	120; 61[*]	male	Byrom & Krebs 1999; Nowak 1991

#: maximum dispersal distance
*: median dispersal distance
^: not included in phylogenetic analysis

Sources:

Anderson, A.E. & Wallmo, O.C. 1984. *Odocoileus hemionus. Mammalian Species*, **219**, 1-9.

Andrews, R. D. & Boggess, E. K. 1978. Ecology of coyotes in Iowa. In: *Coyotes: biology, behavior, and management.* (Ed. by M. Bekoff), pp. 249-265. New York: Academic Press.

Arthur, S. M., Paragi, T. F., & Krohn, W. B. 1993. Dispersal of juvenile fishers in Maine. *Journal of Wildlife Management*, **57**, 868-874.

Ballard, W. B., Ayres, L. A., Krausman, P. R., Reed, D. J., & Fancy, S. G. 1997. Ecology of wolves in relation to a migratory caribou herd in northwest Alaska. *Wildlife Monographs*, **135**, 5-47.

Bekoff, M. 1977. *Canis latrans*. *Mammalian Species*, **79**, 1-9.

Bowen, W. D. 1982. Determining age of coyotes, *Canis latrans*, by tooth sections and tooth-wear patterns. *Canadian Field Naturalist*, **96**, 339-341.

Brockelman, W. Y., Reichard, U., Treesucon, U., & Raemaekers, J. J. 1998. Dispersal, pair formation and social structure in gibbons (*Hylobates lar*). *Behavioral Ecology and Sociobiology*, **42**, 329-339.

Bunnell, F. L. & Harstad, A. S. 1983. Dispersal and dispersion of black-tailed deer: models and observations. *Journal of Mammalogy*, **64**, 201-209.

Byrom, A. E. 2002. Dispersal and survival of juvenile feral ferrets *Mustela furo* in New Zealand. *Journal of Applied Ecology*, **39**, 67-78.

Byrom, A. E. & Krebs, C. J. 1999. Natal dispersal of juvenile arctic ground squirrels in the boreal forest. *Canadian Journal of Zoology-Revue Canadienne de Zoologie*, **77**, 1048-1059.

Caley, M. J. 1987. Dispersal and inbreeding avoidance in muskrats. *Animal Behaviour*, **35**, 1225-1233.

Chapman, J. A. & Tretheway, D. E. C. 1972. Movements within a population of introduced eastern cottontail rabbits. *Journal of Wildlife Management*, **36**, 155-158.

Chapman, J. A., Hockman, J. G., & Ojeda C., M. M. 1980. *Sylvilagus floridanus*. *Mammalian Species*, **136**, 1-8.

Cordes, C. L. & Barkalow, F. S. Jr. 1972. Home range and dispersal in a North Carolina gray squirrel population. *Proceedings of the Southeastern Association of Game and Fish Commissioners*, **26**, 124-135.

Currier, M. J. P. 1983. *Felis concolor*. *Mammalian Species*, 200, 1-7.

Daly, J. C. & Patton, J. L. 1990. Dispersal, gene flow, and allelic diversity between local populations of *Thomomys bottae* pocket gophers in the coastal ranges of California. *Evolution*, **44**, 1283-1294.

Dice, L. R., & Howard, W. E. 1951. Distance of dispersal by prairie deermice from birthplaces to breeding sites. *Contributions from the Laboratory of Vertebrate Biology*, **50**, 1-15.

Dobson, F. S., Smith, A. T., & Gao, W. X. 1998. Social and ecological influences on dispersal and philopatry in the plateau pika (*Ochotona curzoniae*). *Behavioral Ecology*, **9**, 622-635.

Dusek, G. L., Mackie, R. J., Herriges, J. D., Jr., & Compton, B. B. 1989. Population ecology of white-tailed deer along the lower Yellowstone River. *Wildlife Monographs*, **104**, 1-68.

Elliott, L. 1978. Social behavior and foraging ecology of the eastern chipmunk (*Tamias striatus*) in the Adirondack Mountains. *Smithsonian Contributions to Zoology*, **265**, 1-107.

Erlinge, S. 1977. Spacing strategy in stoat *Mustela erminea*. *Oikos*, **28**, 32-42.

Errington, P. L. 1944. Additional studies on tagged young muskrats. *Journal of Wildlife Management*, **8**, 300-306.

Errington, P. L. & Errington, C. S. 1937. Experimental tagging of young muskrats for purpose of study. *Journal of Wildlife Management*, **1**, 49-61.

Fritzell, E. K. & Haroldson, K. J. 1982. *Urocyon cinereoargenteus*. *Mammalian Species*, **189**, 1-8.

Gehrt, S. D. & Fritzell, E. K. 1998. Duration of familial bonds and dispersal patterns for raccoons in south Texas. *Journal of Mammalogy*, **79**, 859-872.

Gese, E. M. & Mech, L. D. 1991. Dispersal of wolves (*Canis lupus*) in northeastern Minnesota, 1969-1989. *Canadian Journal of Zoology-Revue Canadienne de Zoologie*, **69**, 2946-2955.

Gillette, L. N. 1980. Movement patterns of radio-tagged opossums in Wisconsin. *American Midland Naturalist*, **104**, 1-12.

Glenn, L. P. & Miller, L. H. 1980. Seasonal movements of an Alaska peninsula brown bear population. In: *Bears: Their Biology and Management : A Selection of Papers from the Fourth International Conference on Bear Research and Management, Kalispell, Montana, USA, February 1977*. (Ed. Martinka, C. J. & MacArthur, K. L.), pp. 307-312, Tonto Basin, AZ: The Bear Biology Association.

Hackett, D. F. 1987. Dispersal of yearling Columbian ground squirrels. Ph.D. thesis, University of Alberta.

Harrison, R. L. 1992. Dispersal characteristics of juvenile coyotes in Maine. *Journal of Wildlife Management*, **56**, 128-138.

Herrera, E. A. 1992. Growth and dispersal of capybaras (*Hydrochaeris hydrochaeris*) in the llanos of Venezuela. *Journal of Zoology*, **228**, 307-316.

Herrera, E. A. & McDonald, D. W. 1993. Aggression, dominance, and mating success among capybara males (*Hydrochaeris hydrochaeris*). *Behavioral Ecology*, **4**, 114-119.

Hoogland, J. L. 1999. Philopatry, dispersal, and social organization of Gunnison's prairie dogs. *Journal of Mammalogy*, **80**, 243-251.

Jacquot, J. J. & Vessey, S. H. 1995. Influence of the natal environment on dispersal of white-footed mice. *Behavioral Ecology and Sociobiology*, **37**, 407-412.

Jenkins, S.H. & Busher, P. E. 1979. *Castor canadensis*. *Mammalian Species*, **120**, 1-8.

Jensen, B. 1973. Movements of the red fox (*Vulpes vulpes* L.) in Denmark investigated by marking and recovery. *Danish Review of Game Biology*, **8**, 3-20.

Jones, W. T. 1987. Dispersal patterns in kangaroo rats (*Dipodomys spectabilis*). In: *Mammalian Dispersal Patterns: The effects of social structure on population genetics*. (Ed. by Chepko-Sade, B. D. & Tang Halpin, Z.), pp. 119-127. Chicago: University of Chicago Press.

Jones, W. T. 1989. Dispersal distance and the range of nightly movements in Merriam's kangaroo rat. *Journal of Mammalogy*, **70**, 27-34.

Jones, W. T., Waser, P. M., Elliott, L. F., Link, N. E., & Bush, B. B. 1988. Philopatry, dispersal, and habitat saturation in the banner-tailed kangaroo rat, *Dipodomys spectabilis*. *Ecology*, **69**, 1466-1473.

Keane, B. 1990. Dispersal and inbreeding avoidance in the white-footed mouse, *Peromyscus leucopus*. *Animal Behaviour*, **40**, 143-152.

King, C. M. 1983. *Mustela erminea*. *Mammalian Species,* **195**, 1-8.

Lackey, J. A., Huckaby, D. G., & Ormiston, B. G. 1985. *Peromyscus leucopus*. *Mammalian Species*, **247**, 1-10.

Lambin, X. 1994. Natal philopatry, competition for resources, and inbreeding avoidance in Townsend's voles (*Microtus townsendii*). *Ecology*, **75**, 224-235.

Larivière, S. 2001. *Ursus americanus*. *Mammalian Species*, **647**, 1-11.

Larivière, S. & Pasitschniak-Arts, M. 1996. *Vulpes vulpes*. *Mammalian Species*, **537**, 1-11.

Larsen, K. W. & Boutin, S. 1994. Movements, survival, and settlement of red squirrel (*Tamiasciurus hudsonicus*) offspring. *Ecology*, **75**, 214-223.

Leighton, D.R. 1987. Gibbons: Territoriality and Monogamy. In: *Primate Societies*. (Ed. by Smuts, B. B., Cheney, D. L., Seyfarth, R. M., Wrangham, R. W. & Struhsaker, T. T.). Chicago: University of Chicago Press.

Lindzey, F. G. 1978. Movement patterns of badgers in northwestern Utah. *Journal of Wildlife Management*, **42**, 418-422.

Loew, S. S. 1999. Sex-biased dispersal in eastern chipmunks, *Tamias striatus*. *Evolutionary Ecology*, **13**, 557-577.

Long, C. A. 1973. *Taxidea taxus*. *Mammalian Species*, **26**, 1-4.

Maehr, D. S., Land, E. D., Shindle, D. B., Bass, O. L., & Hoctor, T. S. 2002. Florida panther dispersal and conservation. *Biological Conservation*, **106**, 187-197.

McGuire, B., Getz, L. L., Hofmann, J. E., Pizzuto, T., & Frase, B. 1993. Natal dispersal and philopatry in prairie voles (*Microtus ochrogaster*) in relation to population density, season, and natal social environment. *Behavioral Ecology and Sociobiology*, **32**, 293-302.

McLellan, B. N. & Hovey, F. W. 2001. Natal dispersal of grizzly bears. *Canadian Journal of Zoology-Revue Canadienne de Zoologie*, **79**, 838-844.

McNutt, J. W. 1996. Sex-biased dispersal in African wild dogs, *Lycaon pictus*. *Animal Behaviour*, **52**, 1067-1077.

Messick, J. P. & Hornocker, M. G. 1981. Ecology of the badger in southwestern Idaho. *Wildlife Monographs*, **76**, 3-53.

Mitchell, J. L. 1961. Mink movements and populations on a Montana river. *Journal of Wildlife Management*, **25**, 48-54.

Murie, J. O. & Harris, M. A. 1984. The history of individuals in a population of Columbian ground squirrels: source, settlement, and site attachment. In: *The Biology of Ground-Dwelling Squirrels: Annual Cycles, Behavioral Ecology, and Sociality*. (Ed. by Murie, J. O. & Michener, G.R.). Lincoln: University of Nebraska Press.

Nowak, R. M. 1991. *Walker's Mammals of the World*. 5th ed. Baltimore: Johns Hopkins University Press.

Pasitschniak-Arts, M. 1993. *Ursus arctos*. *Mammalian Species*, **439**, 1-10.

Pearson, A. M. 1972. Population characteristics of the northern interior grizzly in the Yukon Territory, Canada. In: *Bears: Their biology and management, Vol. 2. A Selection of Papers from the Second International Conference on Bear Research and Management, Calgary, Alberta, Canada, 6-9 November 1970*. pp. 32-35. Morges: Switzerland: International Union for Conservation of Nature and Natural Resources.

Price, M. V., Kelly, P. A., & Goldingay, R. L. 1994. Distances moved by Stephens kangaroo rat (*Dipodomys stephensi merriam*) and implications for conservation. *Journal of Mammalogy*, **75**, 929-939.

Radespiel, L., Lutermann, H., Schmelting, B., Bruford, M. W., & Zimmerman, E. 2003. Patterns and dynamics of sex-biased dispersal in a nocturnal primate, the grey mouse lemur, *Microcebus murinus*. *Animal Behaviour*, **65**, 709-719.

Randall, J. A. 1993. Behavioral adaptations of desert rodents (Heteromyidae). *Animal Behaviour*, **45**, 263-287.

Ribble, D. O. 1992. Dispersal in a monogamous rodent, *Peromyscus californicus*. *Ecology*, **73**, 859-866.

Richardson, B. J., Hayes, R. A., Wheeler, S. H., & Yardin, M. R. 2002. Social structures, genetic structures and dispersal strategies in Australian rabbit (*Oryctolagus cuniculus*) populations. *Behavioral Ecology and Sociobiology*, **51**, 113-121.

Rogers, L. L. 1987. Factors influencing dispersal in the black bear. In: *Mammalian Dispersal Patterns: The effects of social structure on population genetics*. (Ed. by Chepko-Sade, B. D. & Tang Halpin, Z.), pp. 75-84. Chicago: University of Chicago Press.

Rood, J. P. 1987. Dispersal and intergroup transfer in the dwarf mongoose. In: *Mammalian Dispersal Patterns: The effects of social structure on population genetics*. (Ed. by Chepko-Sade, B. D. & Tang Halpin, Z.), pp. 85-103. Chicago: University of Chicago Press.

Rosatte, R. C. & Gunson, J. R. 1984. Dispersal and home ranges of striped skunks, *Mephitis mephitis*, in an area of population reduction in southern Alberta. *Canadian Field Naturalist*, **98**, 315-319.

Roy, L. D. & Dorrance, M. J. 1985. Coyote movements, habitat use, and vulnerability in central Alberta. *Journal of Wildlife Management*, **49**, 307-313.

Sandell, M., Agrell, J., Erlinge, S., & Nelson, J. 1990. Natal dispersal in relation to population density and sex ratio in the field vole, *Microtus agrestis*. *Oecologia*, **83**, 145-149.

Schmidt, K. 1998. Maternal behaviour and juvenile dispersal in the Eurasian lynx. *Acta Theriologica*, **43**, 391-408.

Soderquist, T. R. & Lill, A. 1995. Natal dispersal and philopatry in the carnivorous marsupial *Phascogale tapoatafa* (Dasyuridae). *Ethology*, **99**, 297-312.

Storm, G. L., Andrews, R. D., Phillips, R. L., Bishop, R. A., Siniff, D. B., & Tester, J. R. 1976. Morphology, reproduction, dispersal, and mortality of Midwestern red fox populations. *Wildlife Monographs*, **49**, 3-82.

Stuewer, F. W. 1943. Reproduction of raccoons in Michigan. *Journal of Wildlife Management*, **7**, 60-73.

Sun, L. X., Muller-Schwarze, D, & Schulte, B. A. 2000. Dispersal pattern and effective population size of the beaver. *Canadian Journal of Zoology-Revue Canadienne de Zoologie*, **78**, 393-398.

Swilling, W. R. & Wooten, M. C. 2002. Subadult dispersal in a monogamous species: the Alabama beach mouse (*Peromyscus polionotus ammobates*). *Journal of Mammalogy*, **83**, 252-259.

Tullar, B. F., & Berchielli, L. T. 1980. Movement of the red fox in central New York. *New York Fish and Game Journal*, **27**, 179-204.

Tullar, B. F., & Berchielli, L. T. 1982. Comparison of red foxes and gray foxes in central New York with respect to certain features of behavior, movement, and mortality. *New York Fish and Game Journal*, **29**, 127-133.

Vangen, K. M., Persson, J., Landa, A., Andersen, R., & Segerstrom, P. 2001. Characteristics of dispersal in wolverines. *Canadian Journal of Zoology-Revue Canadienne de Zoologie*, **79**, 1641-1649.

Van Vuren, D. 1990. Dispersal of yellow-bellied marmots. Ph.D. thesis, University of Kansas.

Wauters, L., & Dhondt, A. A. 1993. Immigration pattern and success in red squirrels. *Behavioral Ecology and Sociobiology*, **33**, 159-167.

Willner, G. R., Feldhamer, G. A., Zucker, E. E.., & Chapman, J. A. 1980. *Ondatra zibethicus*. *Mammalian Species*, **141**, 1-8.

Wilson, D. E. 1982. Wolverine. In: *Wild Mammals of North America: Biology, Management, and Economics*. (Ed. by Chapman, J. A. & Feldhamer, G. A.) Baltimore: Johns Hopkins University Press.
