# Supplementary material for: Social Mating System and Sex-Biased Dispersal in Mammals and Birds: A Phylogenetic Analysis
Source: PLoS One. 2013 Mar 6;8(3):e57980. doi: 10.1371/journal.pone.0057980 (PMC3590276; doi:10.1371/journal.pone.0057980)
Supplement: Table S2 — Mating system and dispersal data for birds. (PDF) [file pone.0057980.s002.pdf]

Table S2: mating system and dispersal data for birds.

taxonomy	Latin name	common name	mating system	male dispersal distance (m)	female dispersal distance (m)	dispersal sex-bias	sources
Anseriformes							
Anatidae	*Branta canadensis*	Canada goose	monogamy	15,200	3,100	male	Lessels 1985
	Cygnus olor	mute swan	monogamy	12,859	7,754	male	Ciaranca et al. 1997; Coleman & Minton 1979
Ciconiiformes							
Accipitridae	*Accipiter cooperii*	Cooper's hawk	monogamy	12,000	14,400	female	Curtis et al. 2006; Rosenfield & Bielefeldt 1992
	Accipiter gentilis	goshawk	monogamy	27,741	15,588	male	Kenward et al. 1993; Squires & Reynolds 1997
	Accipiter nisus	sparrow hawk	monogamy	5,320; 14,000*; 185,000#	9,680; 27,000*; 265,000#	female	Brown & Amadon 1968; Newton & Marquiss 1983
	Aquila adalberti	Spanish imperial eagle	monogamy	123,600	140,600	female	Cramp 1980; Ferrer 1993
	Circus cyaneus	hen harrier	non-monogamy	6,300	5,680	male	Picozzi 1984
	Hieraaetus fasciatus	Bonelli's eagle	monogamy	101,000	189,000	female	Brown & Amadon 1968; Real & Mañosa 2001
	Milvus milvus	red kite	monogamy	7,000*	8,000*	female	Newton et al. 1989
Charadriidae	*Charadrius melodus*	piping plover	monogamy	6,747; 2,525*	7,003; 3,213*	female	Haig & Oring 1988; Wilcox 1959
	Recurvirostra americana	American avocet	monogamy	1,100; 620*	7,300; 6,300*	female	Robinson et al. 1997; Robinson & Oring 1997
Falconidae	*Falco peregrinus*	peregrine falcon	monogamy	58,000*	83,000*	female	Mearns & Newton 1984; White et al. 2002
	Falco sparverius	American kestrel	monogamy	8,280; 4,380*	8,600; 5,060*	female	Miller & Smallwood 1997; Smallwood & Bird 2002

	Gaviidae	*Gavia stellata*	red-throated loon	monogamy	2,170	37,790	female	Barr et al. 2000; Okill 1992
	Procellariidae	*Calonectris diomedea*	Cory's shearwater	monogamy	36; 8*	155; 375*	female	Cramp 1978; Thibault 1993
		Phoebastria immutabilis	Laysan albatross	monogamy	18; 13*; 51#	25; 24*; 43#	female	Awkerman et al. 2009; Fisher 1971
	Scolopacidae	*Calidris alpina*	dunlin	monogamy	1,683; 1,250*	2,101; 2,036*	female	Jackson, D. B. 1994; Soikkeli 1970; Warnock & Gill 1996
		Calidris pusilla	semipalmated sandpiper	monogamy	549*	204*	male	Gratto et al. 1985; Hicklin et al. 2010
		Limosa limosa	black-tailed godwit	monogamy	873	1,080	female	Groen 1993
		Tringa totanus	redshank	monogamy	630	2,200	female	Jackson, D. B. 1994
	Sulidae	*Sula nebouxii*	blue-footed booby	monogamy	33; 24*	41; 28*	female	Nelson 1978; Osorio-Beristain & Drummond 1993
Galliformes								
	Phasianidae	*Bonasa umbellus*	ruffed grouse	non-monogamy	2,140	4,820	female	Rusch et al. 2000; Small & Rusch 1989
		Centrocercus urophasianus	sage grouse	non-monogamy	7,400*	8,800*	female	Dunn & Braun 1985; Schroeder et al. 1999
		Dendragapus obscurus	blue grouse	non-monogamy	1,100*	2,000*	female	Hines 1986; Jamieson & Zwickel 1983; Zwickel & Bendell 2005
		Lagopus lagopus	willow ptarmigan	monogamy	1,000*; 4,000#	2,900*; 7,500#	female	Hannon et al. 1998; Martin & Hannon 1987
		Lagopus leucurus	white-tailed ptarmigan	non-monogamy	1,250*; 7,500#	4,000*; 29,000#	female	Braun et al. 1993; Giesen & Braun 1993
		Tetrao tetrix	black grouse	non-monogamy	1,500; 800*	8,000; 7,300*	female	Caizergues & Ellison 2002; Cramp 1980
	Gruidae	*Grus canadensis*	Florida sandhill crane	monogamy	3,940	11,580	female	Nesbitt et al. 2002
Passeriformes								
	Certhiidae	*Troglodytes aedon*	house wren	monogamy	608*	674*	female	Drilling & Thompson 1988; Johnson 1998
	Cinclidae	*Cinclus cinclus*	Welsh dipper	monogamy	3,090; 3,000*	6,450; 5,000*	female	Cramp & Simmons 1988; Tyler et al. 1990
	Corvidae	*Aphelocoma coerulescens*	Florida scrub jay	monogamy	387; 170*; 4,068#	1,165; 678*; 6,102#	female	Woolfenden & Fitzpatrick 1978; Woolfenden & Fitzpatrick 1996
		Perisoreus canadensis	gray jay	monogamy	3	7	female	Strickland 1991; Strickland & Ouellet 2011

	Pica pica	magpie	monogamy	358; 350*; 1,300#	465; 498*; 800#	female	Birkhead et al. 1986; Eden 1987; Trost 1999
Fringillidae	*Dendroica kirtlandii*	Kirtland's warbler	monogamy	5,601	21,320	female	Berger & Radabaugh 1968; Mayfield 1992
	Emberiza schoeniclus	reed bunting	non-monogamy	1,241; 1,000*	1,864; 1,500*	female	Cramp et al. 1994; Haukioja, 1971
	Melospiza melodia	song sparrow	monogamy	110*	127*	female	Arcese 1989
	Passerculus sandwichensis	Savannah sparrow	non-monogamy	262; 202*; 1,381#	309; 248*; 1,540#	female	Wheelwright & Mauck 1998; Wheelwright & Rising 2008
	Passerina cyanea	indigo bunting	monogamy	870	708	male	Payne 1991; Payne 2006
	Zonotrichia leucophrys	white-crowned sparrow	monogamy	555; 376*	614; 526*	female	Baker & Mewaldt 1978; Chilton et al. 1995
Hirundinidae	*Hirundo rustica*	barn swallow	monogamy	6,375*	8,125*	female	Brown & Brown 1999; Shields 1982
Maluridae	*Malurus pulcherrimus*	blue-breasted fairy wren	monogamy	219	319	female	Rowley & Russell 2002
	Malurus splendens	splendid fairy wren	monogamy	100; 2,400#	200; 2,400#	female	Russell & Rowley 1993
Muscicapidae	*Ficedula albicollis*	collared flycatcher	monogamy	518	840	female	Cramp & Perrins 1993; Pärt 1990
	Ficedula hypoleuca	pied flycatcher	monogamy	445	600	female	Cramp & Perrins 1993; Potti & Montalvo 1991
	Saxicola rubetra	whinchat	monogamy	500	500	equal between the sexes	Bastian 1992; Cramp & Simmons 1988
	Sialia sialis	eastern bluebird	monogamy	1,080; 570*; 8,170#	1,390; 900*; 8,690#	female	Plissner & Gowaty 1996
Paridae	*Parus ater*	coal tit	monogamy	525	570	female	Cramp & Perrins 1993; Dietrich et al. 2003
	Parus palustris	marsh tit	monogamy	1,100*; 4,600#	2,600*; 7,300#	female	Cramp & Perrins 1993; Nilsson 1989
Sittidae	*Sitta europaea*	nuthatch	monogamy	1,120*; 6,100#	800*; 11,300#	male	Matthysen & Schmidt 1987
Sylviidae	*Chamaea fasciata*	wrentit	monogamy	373	368	male	Baker et al. 1995; Sibley 2009
	Locustella pleskei	Styan's grasshopper warbler	monogamy	68	74	female	Nagata 1993

	Phylloscopus trochilus	willow warbler	monogamy	893; 760*	580; 580*	male	Lawn 1982; Tianinen 1983
Piciformes							
Picidae	*Melanerpes formicivorus*	acorn woodpecker	non-monogamy	220	530	female	Koenig et al. 1995; Koenig et al. 2000
	Picoides borealis	red-cockaded woodpecker	monogamy	2089*	3689*	female	Jackson, J. A. 1994; Schiegg et al. 2006
Strigiformes							
Strigidae	*Aegolius funereus*	boreal owl	monogamy	21,000*	88,000*	female	Hayward & Hayward 1993; Korpimäki et al. 1987
	Athene cunicularis	little owl	monogamy	414*	1,116*	female	Millsap & Bear 1997; Poulin et a. 2011

#: maximum dispersal distance
*: median dispersal distance

Sources:

Arcese, P. 1989. Intrasexual competition, mating system and natal dispersal in song sparrows. *Animal Behaviour*, **38**, 958-979.

Awkerman, J., Anderson, D. & Whittow, G. C. 2009. Laysan Albatross (*Phoebastria immutabilis*), The Birds of North America Online (A. Poole, Ed.). Ithaca: Cornell Lab of Ornithology; Retrieved from the Birds of North America Online: http://bna.birds.cornell.edu/bna/species/066. doi:10.2173/bna.66

Baker, M., Nur, N. & Geupel, G. R. 1995. Correcting biased estimates of dispersal and survival due to limited study area: theory and an application using wrentits. *Condor*, **97**, 663-674.

Baker, M. C. & Mewaldt, L. R. 1978. Song dialects as barriers to dispersal in White-Crowned Sparrows, *Zonotrichia leucophrys nuttalli*. *Evolution*, **32**, 712-722.

Barr, J. F., Eberl, C. & McIntyre, J. W. 2000. Red-throated Loon (*Gavia stellata*), The Birds of North America Online (A. Poole, Ed.). Ithaca: Cornell Lab of Ornithology; Retrieved from the Birds of North America Online: http://bna.birds.cornell.edu/bna/species/513. doi:10.2173/bna.513

Bastian, H. V. 1992. Breeding and natal dispersal of Whinchats *Saxicola rubetra*. *Ringing and Migration*, **13**, 13-19.

Berger, A. J. & Radabaugh, B. E. 1968. Returns of Kirtland's Warblers to the breeding grounds. *Bird-banding*, **39**, 161-186.

Birkhead, T. R., Eden, S. F., Clarkson, K., Goodburn, S. F., & Pellatt, J. 1986. Social organization of a population of magpies *Pica pica*. *Ardea*, **74**, 59-68.

Braun, C. E., Martin, K. & Robb, L. A. 1993. White-tailed Ptarmigan (*Lagopus leucura*), The Birds of North America Online (A. Poole, Ed.). Ithaca: Cornell Lab of Ornithology; Retrieved from the Birds of North America Online: http://bna.birds.cornell.edu/bna/species/068. doi:10.2173/bna.68

Brown, C. R. and Brown, M. B. 1999. Barn Swallow (*Hirundo rustica*), The Birds of North America Online (A. Poole, Ed.). Ithaca: Cornell Lab of Ornithology; Retrieved from the Birds of North America Online: http://bna.birds.cornell.edu/bna/species/452. doi:10.2173/bna.452

Brown, L. & Amadon, D. 1968. Eagles, Hawks, and Falcons of the World, Vol. 2. New York: McGraw-Hill.

Caizergues, A. & Ellison, L. N. 2002. Natal dispersal and its consequences in Black Grouse *Tetrao tetrix*. *Ibis*, **144**, 478-487.

Chilton, G., Baker, M. C., Barrentine, C. D. & Cunningham, M. A. 1995. White-crowned Sparrow (*Zonotrichia leucophrys*), The Birds of North America Online (A. Poole, Ed.). Ithaca: Cornell Lab of Ornithology; Retrieved from the Birds of North America Online: http://bna.birds.cornell.edu/bna/species/183. doi:10.2173/bna.183

Ciaranca, M. A., Allin, C. C., & Jones, G. S. 1997. Mute Swan (*Cygnus olor*). The Birds of North America Online. (Ed: Poole, A.). Ithaca: Cornell Lab of Ornithology; Retrieved from the Birds of North America Online: http://bna.birds.cornell.edu/bna/species/273. doi:10.2173/bna.273.

Coleman, A. E. & Minton, C. D. 1979. Pairing and breeding of Mute Swans in relation to natal area. *Wildfowl*, **30**, 27-30.

Cramp, S. 1978. Handbook of the Birds of Europe, the Middle East, and North Africa: The Birds of the Western Palearctic Volume I: Ostrich to Ducks. New York: Oxford University Press.

Cramp, S. 1980. Handbook of the Birds of Europe, the Middle East and North Africa: The Birds of the Western Paleartic, Vol. 2: Hawks to Bustards. New York: Oxford University Press.

Cramp, S. & Perrins, C. M. 1993. Handbook of the Birds of Europe, the Middle East, and North Africa: The Birds of the Western Palearctic Volume VII: Flycatchers to Shrikes. New York: Oxford University Press.

Cramp, S., Perrins, C. M., & Brooks, D. J. 1994. Handbook of the Birds of Europe, the Middle East, and North Africa: The Birds of the Western Palearctic Volume IX: Buntings and New World Warblers. New York: Oxford University Press.

Cramp, S. & Simmons, K. E. L. 1988. Handbook of the Birds of Europe, the Middle East and North Africa: The Birds of the Western Palearctic Volume V: Tyrant Flycatchers to Thrushes. New York: Oxford University Press.

Curtis, O. E., Rosenfield, R. N. & Bielefeldt, J. 2006. Cooper's Hawk (*Accipiter cooperii*), The Birds of North America Online (A. Poole, Ed.). Ithaca: Cornell Lab of Ornithology; Retrieved from the Birds of North America Online: http://bna.birds.cornell.edu/bna/species/075. doi:10.2173/bna.75

Dietrich, V. C. J., Schmoll, T., Winkel, W., & Lubjuhn, T. 2003. Survival to first breeding is not sex-specific in the Coal Tit (*Parus ater*). *Journal für Ornithologie*, **144**, 148-156.

Drilling, N. E. & Thompson, C. F. 1988. Natal and breeding dispersal in house wrens (*Troglodytes aedon*). *Auk*, **105**, 480-491.

Dunn, P. O. & Braun, C. E. 1985. Natal dispersal and lek fidelity of sage grouse. *Auk*, **102**, 621-627.

Eden, S. F. 1987. Natal philopatry of the magpie *Pica pica*. *Ibis*, **129**, 477-490.

Ferrer, M. 1993. Ontogeny of dispersal distances in young Spanish imperial eagles. *Behavioral Ecology and Sociobiology*, **32**, 259-263.

Fisher, H. I. 1971. Experiments on homing in Laysan albatrosses, *Diomedea immutabilis*. *Condor*, **73**, 389-400.

Giesen, K. M. & Braun, C. E. 1993. Natal dispersal and recruitment of juvenile white-tailed Ptarmigan in Colorado. *Journal of Wildlife Management*, **57**, 72-77.

Gratto, C. L., Morrison, R. I. G., & Cooke, F. 1985. Philopatry, site tenacity, and mate fidelity in the Semipalmated Sandpiper. *Auk*, **102**, 16-24.

Groen, N. A. 1993. Breeding site tenacity and natal philopatry in the black-tailed Godwit *Limosa limosa*. *Ardea*, **81**, 107-113.

Haig, S. M. & Oring, L. W. 1988. Mate, site, and territory fidelity in piping plovers. *Auk*, **105**, 268-277.

Hannon, S. J., Eason, P. K. & Martin, K. 1998. Willow Ptarmigan (*Lagopus lagopus*), The Birds of North America Online (A. Poole, Ed.). Ithaca: Cornell Lab of Ornithology; Retrieved from the Birds of North America Online: http://bna.birds.cornell.edu/bna/species/369. doi:10.2173/bna.369

Haukioja, E. 1971. Short-distance dispersal in the Reed Bunting *Emberiza schoeniclus*. *Ornis Fennica*, **48**, 45-67.

Hayward, G. D. & Hayward, P. H. 1993. Boreal Owl (*Aegolius funereus*), The Birds of North America Online (A. Poole, Ed.). Ithaca: Cornell Lab of Ornithology; Retrieved from the Birds of North America Online: http://bna.birds.cornell.edu/bna/species/063. doi:10.2173/bna.63

Hicklin, P. & Gratto-Trevor, C. L. 2010. Semipalmated Sandpiper (*Calidris pusilla*), The Birds of North America Online (A. Poole, Ed.). Ithaca: Cornell Lab of Ornithology; Retrieved from the Birds of North America Online: http://bna.birds.cornell.edu/bna/species/006. doi:10.2173/bna.6

Hines, J. E. 1986. Survival and reproduction of dispersing blue grouse. *Condor*, **88**, 43-49.

Jackson, D. B. 1994. Breeding dispersal and site-fidelity in three monogamous wader species in the Western Isles, UK. *Ibis*, **136**, 463-473.

Jackson, J. A. 1994. Red-cockaded Woodpecker (*Picoides borealis*), The Birds of North America Online (A. Poole, Ed.). Ithaca: Cornell Lab of Ornithology; Retrieved from the Birds of North America Online: http://bna.birds.cornell.edu/bna/species/085. doi:10.2173/bna.85

Jamieson, I. G. & Zwickel, F. C. 1983. Dispersal and site fidelity in Blue Grouse. *Canadian Journal of Zoology-Revue Canadienne de Zoologie*, **61**, 570-573.

Johnson, L. S. 1998. House Wren (*Troglodytes aedon*), The Birds of North America Online (A. Poole, Ed.). Ithaca: Cornell Lab of Ornithology; Retrieved from the Birds of North America Online: http://bna.birds.cornell.edu/bna/species/380. doi:10.2173/bna.380

Kenward, R. E., Marcström, V., & Karlbom, M. 1993. Post-nestling behavior in goshawks, *Accipiter gentilis*: II. Sex differences in sociality and nest-switching. *Animal Behaviour*, **46**, 371-378.

Koenig, W. D., Hooge, P. N., Stanback, M. T., & Haydock, J. 2000. Natal dispersal in the cooperatively breeding acorn woodpecker. *Condor*, **102**, 492-502.

Koenig, W. D., Stacey, P. B., Stanback, M. T. & Mumme, R. L. 1995. Acorn Woodpecker (*Melanerpes formicivorus*), The Birds of North America Online (A. Poole, Ed.). Ithaca: Cornell Lab of Ornithology; Retrieved from the Birds of North America Online: http://bna.birds.cornell.edu/bna/species/194. doi:10.2173/bna.194

Korpimäki, E., Lagerström, M., & Saurola, P. 1987. Field evidence for nomadism in Tengmalm's owl *Aegolius funereus*. *Ornis Scandinavica*, **18**, 1-4.

Lawn, M. R. 1982. Pairing systems and site tenacity of the Willow Warbler *Phylloscopus trochilus* in southern England. *Ornis Scandinavica*, **13**, 193-199.

Lessells, C. M. 1985. Natal and breeding dispersal of Canada geese *Branta candensis*. *Ibis*, **127**, 31-41.

Martin, K. & Hannon, S. J. 1987. Natal philopatry and recruitment of willow ptarmigan in north central and northwestern Canada. *Oecologia*, **71**, 518-524.

Matthysen, E. & Schmidt, K. 1987. Natal dispersal in the nuthatch. *Ornis Scandinavica*, **18**, 313-316.

Mayfield, H. F. 1992. Kirtland's Warbler (*Setophaga kirtlandii*), The Birds of North America Online (A. Poole, Ed.). Ithaca: Cornell Lab of Ornithology; Retrieved from the Birds of North America Online: http://bna.birds.cornell.edu/bna/species/019. doi:10.2173/bna.19

Mearns, R. & Newton, I. 1984. Turnover and dispersal in a Peregrin *Falco peregrinus* population. *Ibis*, **126**, 347-355.

Miller, K. E. & Smallwood, J. A. 1997. Natal dispersal and philopatry of southeastern American Kestrels in Florida. *Wilson Bulletin*, **109**, 226-232.

Millsap, B. A. & Bear, C. 1997. Territory fidelity, mate fidelity, and dispersal in an urban population of Florida burrowing owls. *Raptor Research Report*, **9**, 91-98.

Nagata, H. 1993. The structure of a local population and dispersal pattern in the Styan's grasshopper warbler, *Locustella pleskei*. *Ecological Research*, **8**, 1-9.

Nelson, B. 1978: The Sulidae: Gannets and Boobies. New York: Oxford University Press.

Nesbitt, S. A., Schwikert, S. T., & Folk, M. J. 2002. Natal dispersal in Florida sandhill cranes. *Journal of Wildlife Management*, **66**, 349-352.

Newton, I., Davis, P. E., & Davis, J. E. 1989. Age of first breeding, dispersal and survival of Red Kites *Milvus milvus* in Wales. *Ibis*, **131**, 16-21.

Newton, I. & Marquiss, M. 1983. Dispersal of sparrowhawks between birthplace and breeding place. *Journal of Animal Ecology*, **52**, 463-477.

Nilsson, J. 1989. Causes and consequences of natal dispersal in the Marsh Tit, *Parus palustris*. *Journal of Animal Ecology*, **58**, 619-636.

Okill, J. D. 1992. Natal dispersal and breeding site fidelity of Red-throated Divers *Gavia stellata* in Shetland. *Ringing and Migration*, **13**, 57-58.

Osorio-Beristain, M. & Drummond, H. 1993. Natal dispersal and deferred breeding in the Blue-Footed Booby. *Auk*, **110**, 234-239.

Pärt, T. 1990. Dispersal in the collared flycatcher: possible causes and reproductive consequences. *Ornis Scandinavica*, **21**, 83-88.

Payne, R. B. 2006. Indigo Bunting (*Passerina cyanea*), The Birds of North America Online (A. Poole, Ed.). Ithaca: Cornell Lab of Ornithology; Retrieved from the Birds of North America Online: http://bna.birds.cornell.edu/bna/species/004. doi:10.2173/bna.4

Payne, R. B. 1991. Natal dispersal and population structure in a migratory songbird, the Indigo Bunting. *Evolution*, **45**, 49-62.

Picozzi, N. 1984. Breeding biology of polygynous hen harriers *Circus c. cyaneus* in Orkney. *Ornis Scandinavica*, **15**, 1-10.

Plissner, J. H. & Gowaty, P. A. 1996. Patterns of natal dispersal, turnover and dispersal costs in eastern bluebirds. *Animal Behaviour*, **51**, 1307-1322.

Potti, J. & Montalvo, S. 1991. Return rate, age at first breeding and natal dispersal of pied flycatchers *Ficedula hypoleuca* in central Spain. *Ardea*, **79**, 419-428.

Poulin, R., Todd, L. D., Haug, E. A., Millsap, B. A. & Martell, M. S. 2011. Burrowing Owl (*Athene cunicularia*), The Birds of North America Online (A. Poole, Ed.). Ithaca: Cornell Lab of Ornithology; Retrieved from the Birds of North America Online: http://bna.birds.cornell.edu/bna/species/061. doi:10.2173/bna.61

Real, J. & Mañosa, S. 2001. Dispersal of juvenile and immature Bonelli's eagles in northeastern Spain. *Journal of Raptor Research*, **35**, 9-14.

Robinson, J. A. & Oring, L. W. 1997. Natal and breeding dispersal in American Avocets. *Auk*, **114**, 416-430.

Robinson, J. A., Oring, L. W., Skorupa, J. P. & Boettcher, R. 1997. American Avocet (*Recurvirostra americana*), The Birds of North America Online (A. Poole, Ed.). Ithaca: Cornell Lab of Ornithology; Retrieved from the Birds of North America Online: http://bna.birds.cornell.edu/bna/species/275. doi:10.2173/bna.275

Rosenfield, R. N. & Bielefeldt, J. 1992. Natal dispersal and inbreeding in the Cooper's hawk. *Wilson Bulletin*, **104**, 182-184.

Rowley, I. & Russell, E. 2002. A population study of the Blue-breasted Fairy-wren, *Malurus pulcherrimus*, at Dryandra, Western Australia. *Emu*, **102**, 127-135.

Rusch, D. H., Destefano, S., Reynolds, M. C. & Lauten, D. 2000. Ruffed Grouse (*Bonasa umbellus*), The Birds of North America Online (A. Poole, Ed.). Ithaca: Cornell Lab of Ornithology; Retrieved from the Birds of North America Online: http://bna.birds.cornell.edu/bna/species/515. doi:10.2173/bna.515

Russell, E. M. & Rowley, I. 1993. Philopatry or dispersal: competition for territory vacancies in the splendid fairy-wren, *Malurus splendens*. *Animal Behaviour*, **45**, 519-539.

Schiegg, K., Daniels, S. J., Walters, J. R., Priddy, J. A., & Pasinelli, G. 2006. Inbreeding in red-cockaded woodpeckers: effects of natal dispersal distance and territory location. *Biological Conservation*, **131**, 544-552.

Schroeder, M. A., Young, J. R., and Braun, C. E. 1999. Greater Sage-Grouse (*Centrocercus urophasianus*), The Birds of North America Online (A. Poole, Ed.). Ithaca: Cornell Lab of Ornithology; Retrieved from the Birds of North America Online: http://bna.birds.cornell.edu/bna/species/425. doi:10.2173/bna.425

Shields, W. M. 1984. Factors affecting nest and site fidelity in Adirondack Bark Swallows (*Hirundo rustica*). *Auk*, **101**, 780-789.

Sibley, D. A. 2009. The Sibley Guide to Bird Life and Behavior. New York: Knopf.

Small, R. J. & Rusch, D. H. 1989. The natal dispersal of ruffed grouse. *Auk*, **106**, 72-79.

Smallwood, J. A. & Bird, D. M. 2002. American Kestrel (*Falco sparverius*), The Birds of North America Online (A. Poole, Ed.). Ithaca: Cornell Lab of Ornithology; Retrieved from the Birds of North America Online: http://bna.birds.cornell.edu/bna/species/602. doi:10.2173/bna.602

Squires, J. R. & Reynolds, R. T. 1997. Northern Goshawk (*Accipiter gentilis*), The Birds of North America Online (A. Poole, Ed.). Ithaca: Cornell Lab of Ornithology; Retrieved from the Birds of North America Online: http://bna.birds.cornell.edu/bna/species/298. doi:10.2173/bna.298

Soikkeli, M. 1970. Dispersal of Dunlin *Calidris alpina* in relation to sites of birth and breeding. *Ornis Fennica*, **47**, 1-9.

Strickland, D. & Ouellet, H. 2011. Gray Jay (*Perisoreus canadensis*), The Birds of North America Online (A. Poole, Ed.). Ithaca: Cornell Lab of Ornithology; Retrieved from the Birds of North America Online: http://bna.birds.cornell.edu/bna/species/040. doi:10.2173/bna.40

Strickland, D. 1991. Juvenile dispersal in Gray Jays: dominant brood member expels siblings from natal territory. *Canadian Journal of Zoology-Revue Canadienne de Zoologie*, **69**, 2935-2945.

Thibault, J. 1993. Natal philopatry in the Cory's Shearwater (*Calonectris d. diomedea*) on Lavezzi Island, Corsica. *Colonial Waterbirds*, **16**, 77-82.

Tianinen, J. 1983. Dynamics of a local population of the willow warbler *Phylloscopus trochilus* in southern Finland. *Ornis Scandinavica*, **14**, 1-15.

Trost, C. H. 1999. Black-billed Magpie (*Pica hudsonia*), The Birds of North America Online (A. Poole, Ed.). Ithaca: Cornell Lab of Ornithology; Retrieved from the Birds of North America Online: http://bna.birds.cornell.edu/bna/species/389. doi:10.2173/bna.389

Tyler, S. J., Ormerod, S. J., & Lewis, J. M. S. 1990. The post-natal and breeding dispersal of Welsh Dippers *Cinclus cinclus*. *Bird Study*, **37**, 18-23.

Warnock, N. D. & Gill, R. E. 1996. Dunlin (*Calidris alpina*), The Birds of North America Online (A. Poole, Ed.). Ithaca: Cornell Lab of Ornithology; Retrieved from the Birds of North America Online: http://bna.birds.cornell.edu/bna/species/203. doi:10.2173/bna.203

Wheelwright, N. T. & Mauck, R. A. 1998. Philopatry, natal dispersal, and inbreeding avoidance in an island population of Savannah Sparrows. *Ecology*, **79**, 755-767.

Wheelwright, N. T. & Rising, J. D. 2008. Savannah Sparrow (*Passerculus sandwichensis*), The Birds of North America Online (A. Poole, Ed.). Ithaca: Cornell Lab of Ornithology; Retrieved from the Birds of North America Online: http://bna.birds.cornell.edu/bna/species/045. doi:10.2173/bna.45

White, C. M., Clum, N. J., Cade, T. J. & Hunt, W. G. 2002. Peregrine Falcon (*Falco peregrinus*), The Birds of North America Online (A. Poole, Ed.). Ithaca: Cornell Lab of Ornithology; Retrieved from the Birds of North America Online: http://bna.birds.cornell.edu/bna/species/660. doi:10.2173/bna.660

Wilcox, L. R. 1959. A twenty year banding study of the Piping Plover. *Auk*, **76**, 129-152.

Woolfenden, G. E. & Fitzpatrick, J. W. 1996. Florida Scrub-Jay (*Aphelocoma coerulescens*), The Birds of North America Online (A. Poole, Ed.). Ithaca: Cornell Lab of Ornithology; Retrieved from the Birds of North America Online: http://bna.birds.cornell.edu/bna/species/228. doi:10.2173/bna.228

Woolfenden, G. E. & Fitzpatrick, J. W. 1978. The inheritance of territory in group-breeding birds. *BioScience*, **28**, 104-108.

Zwickel, F. C. & Bendell, J. F. 2005. Blue Grouse (*Dendragapus obscurus*), The Birds of North America Online (A. Poole, Ed.). Ithaca: Cornell Lab of Ornithology; Retrieved from the Birds of North America Online: http://bna.birds.cornell.edu/bna/species/015. doi:10.2173/bna.15
